# Author Correction: Rationalizing the light-induced phase separation of mixed halide organic–inorganic perovskites

**DOI:** 10.1038/s41467-017-02224-6

**Published:** 2018-01-11

**Authors:** Sergiu Draguta, Onise Sharia, Seog Joon Yoon, Michael C. Brennan, Yurii V. Morozov, Joseph S. Manser, Prashant V. Kamat, William F. Schneider, Masaru Kuno

**Affiliations:** 1 Department of Chemistry and Biochemistry, University of Notre Dame, Notre Dame, IN 46556 USA; 2 Department of Chemical and Biomolecular Engineering, University of Notre Dame, Notre Dame, IN 46556 USA; 3 Notre Dame Radiation Laboratory, University of Notre Dame, Notre Dame, IN 46556 USA

Correction to: *Nature Communications* 10.1038/s41467-017-00284-2, published online 04 Aug 2017

The original version of this Article contained an error in the spelling of the author Joseph S. Manser, which was incorrectly given as Joseph M. Manser. This has now been corrected in both the PDF and HTML versions of the Article.

