# Supplementary material for: Rationalizing the light-induced phase separation of mixed halide organic–inorganic perovskites
Source: Nat Commun. 2017 Aug 4;8:200. doi: 10.1038/s41467-017-00284-2 (PMC5544754; doi:10.1038/s41467-017-00284-2)
Supplement: Supplementary file 1 — Supplementary Information [file 41467_2017_284_MOESM1_ESM.pdf]

File Name: Supplementary Information
Description: Supplementary Figures, Supplementary Table, Supplementary Notes and Supplementary References

File Name: Peer Review File
Description:

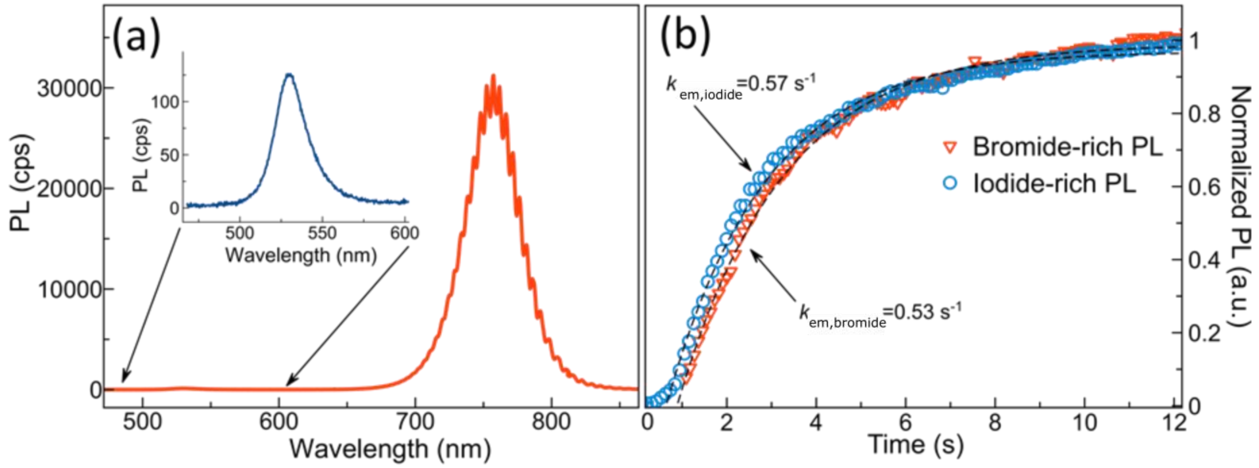

Supplementary Figure 1. Emission spectra and kinetics of iodide-rich and bromide-rich domains during illumination. (a) $MAPb(I_{0.5}Br_{0.5})_3$ ($MA=CH_3NH_3^+$) emission spectra (PL, photoluminescence) after 1 minute of illumination (I_{exc}=60 mW cm^{-2}, λ_{exc}=405 nm). (b) Emission kinetics of iodide-rich and bromide-rich domains during illumination.

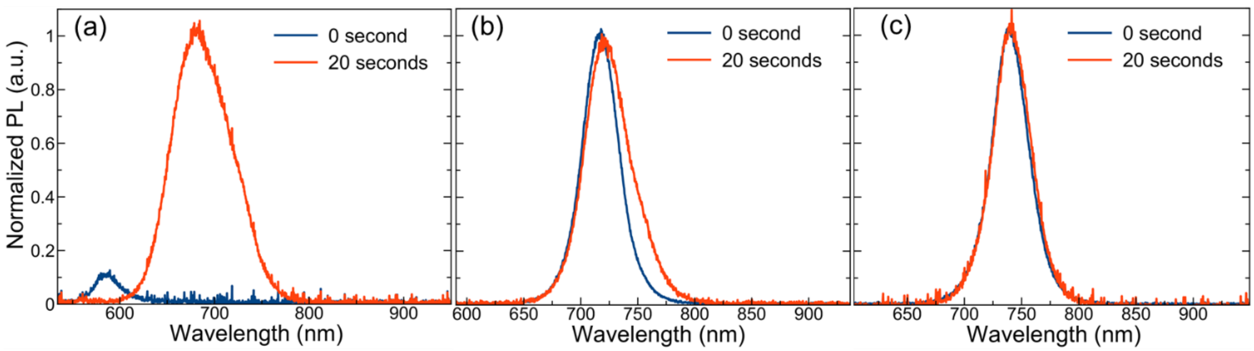

Supplementary Figure 2. Emission spectral changes of $MAPb(I_{1-x}Br_x)_3$ thin films for x=0.12-0.87 under illumination. Emission spectra (PL, photoluminescence) of $MAPb(I_{1-x}Br_x)_3$ ($MA=CH_3NH_3^+$) thin films under I_{exc}=250 mW cm^{-2} for (a) x=0.87, (b) x=0.22, and (c) x=0.12 at 0 and 20 seconds during illumination.

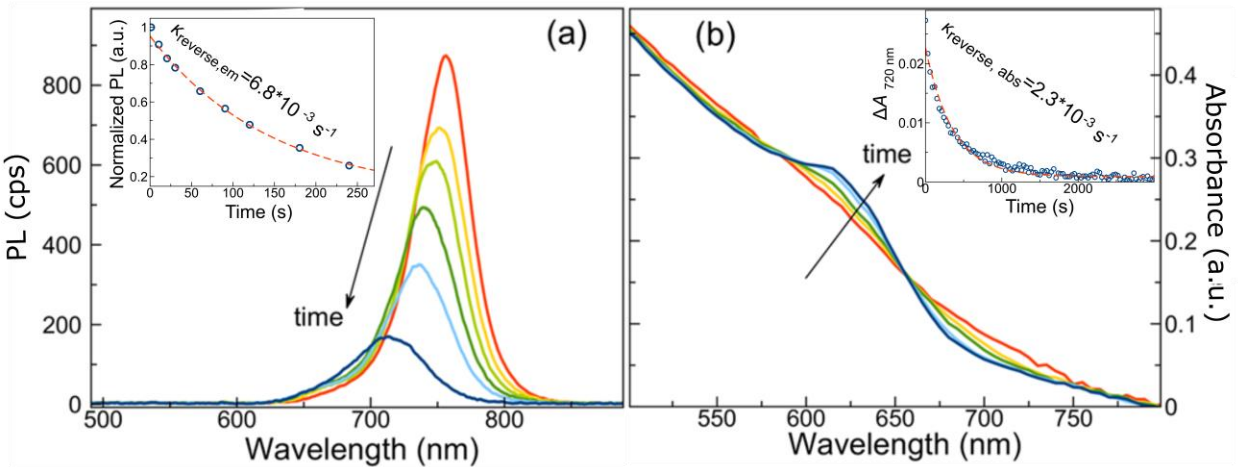

Supplementary Figure 3. Time evolution of emission and absorption spectra of $MAPb(I_{0.5}Br_{0.5})_3$ thin films during recovery. (a) Emission spectra (PL, photoluminescence) of a $MAPb(I_{0.5}Br_{0.5})_3$ ($MA=CH_3NH_3^+$) thin film during recovery after being illuminated for 1 minute with I_{exc}=20 mW cm^{-2}. Inset: Integrated PL decay of the iodide rich phase during recovery. The dashed red line is an exponential decay fit to the data. (b) Absorption spectra of a $MAPb(I_{0.5}Br_{0.5})_3$ thin film during recovery after being illuminated for 1 minute with I_{exc}=25 mW cm^{-2}. Inset: ΔA [$\Delta A = A(0) - A(t)$] (A, absorbance) at 720 nm during recovery. The dashed red line represents an exponential decay fit to the data.

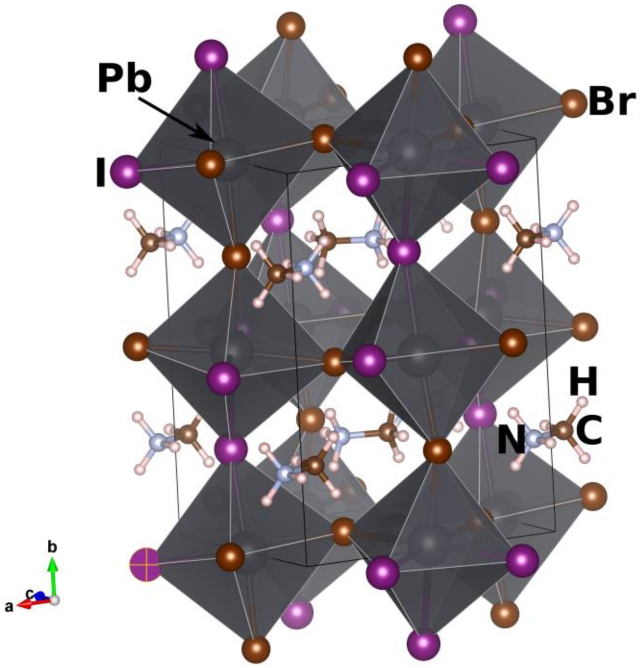

Supplementary Figure 4. Relaxed structure of $MAPb(Br_{0.5}I_{0.5})_3$ ($MA=CH_3NH_3^+$).

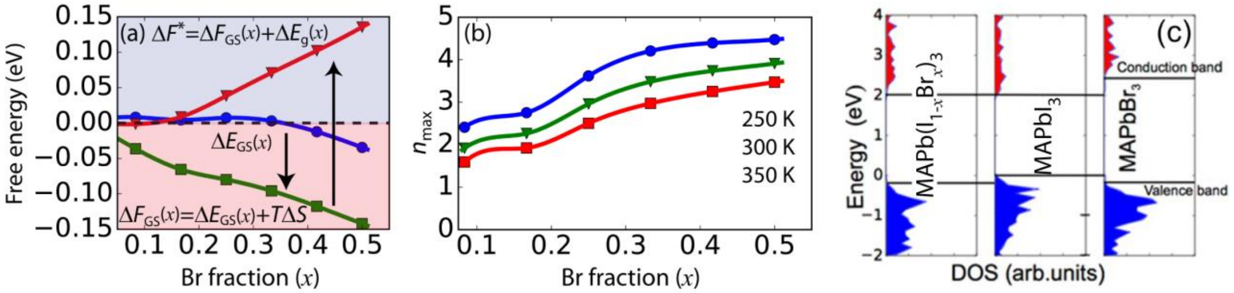

Supplementary Figure 5. Density functional theory modeling of halide phase segregation. (a) Estimated free energy of formation differences as a function of bromide fraction *x*. The blue line/circles are generalized gradient approximation (GGA)-computed 0 K ground state formation energies, including spin-orbit coupling. The green line/squares are free energies of mixing at 300 K, assuming ideal mixing on the halide sublattice. The red line/inverted triangles represent the free energy difference after single photon absorption. All energies are reported per two formula units. Blue and pink shaded areas indicate positive (phase separation favored) and negative (phase separation unfavored) free energy regions. (b) Approximate size of separated domains, n_{max}, following single photon absorption (see Supplementary Equation 17**)**. (c) GGA-computed density of states for $MAPb(I_{0.5}Br_{0.5})_3$, $MAPbI_3$ and $MAPbBr_3$ along with their relative band offsets.

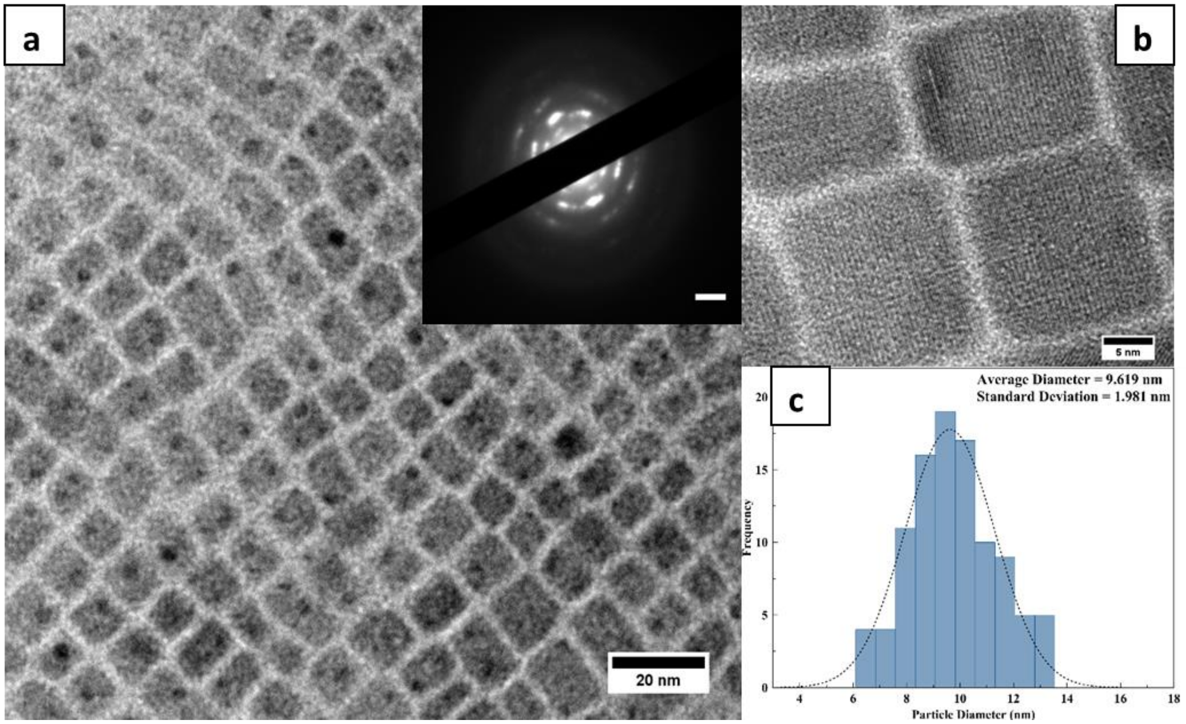

Supplementary Figure 6. Transmission electron microscopy images of $CsPbI_{1.65}Br_{1.35}$ perovskite nanocrystals. (a) Low (scale bar 20 nm) and (b) high (scale bar 5 nm) magnification transmission electron microscopy (TEM) images of $CsPbI_{1.65}Br_{1.35}$ perovskite nanocrystals. The inset in (a) shows the selected area electron diffraction (SAED) pattern of resulting nanocrystals. Scale bar = 0.2 Å^{-1}. (c) TEM-based sizing histogram from where the average nanoparticle diameter was determined to be 9.62 ± 1.98 nm (s.d.).

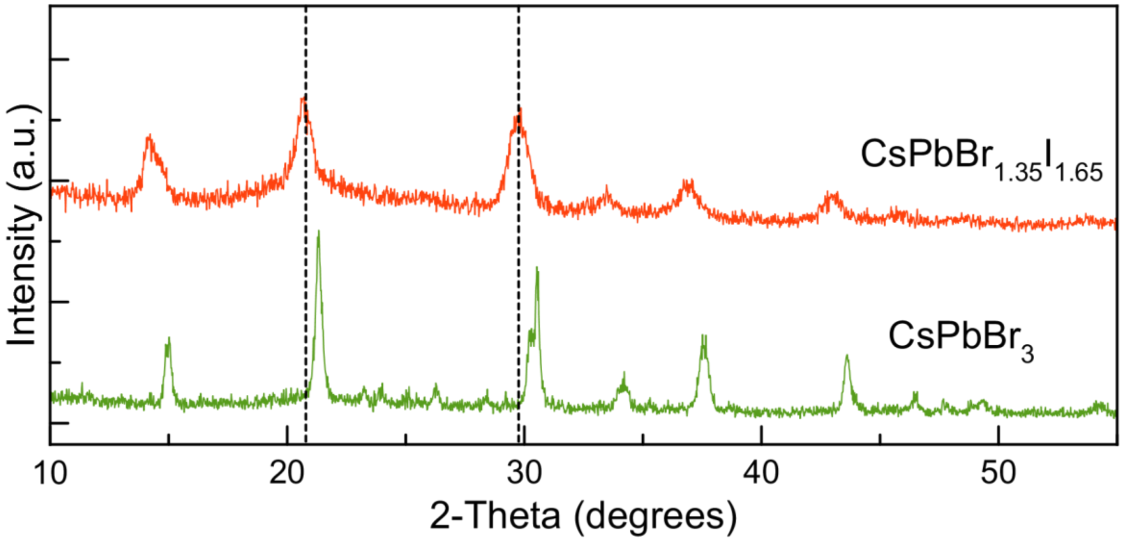

Supplementary Figure 7. Ensemble powder X-ray diffraction (XRD) patterns of $CsPbI_{1.65}Br_{1.35}$ and $CsPbBr_3$ nanocrystals.

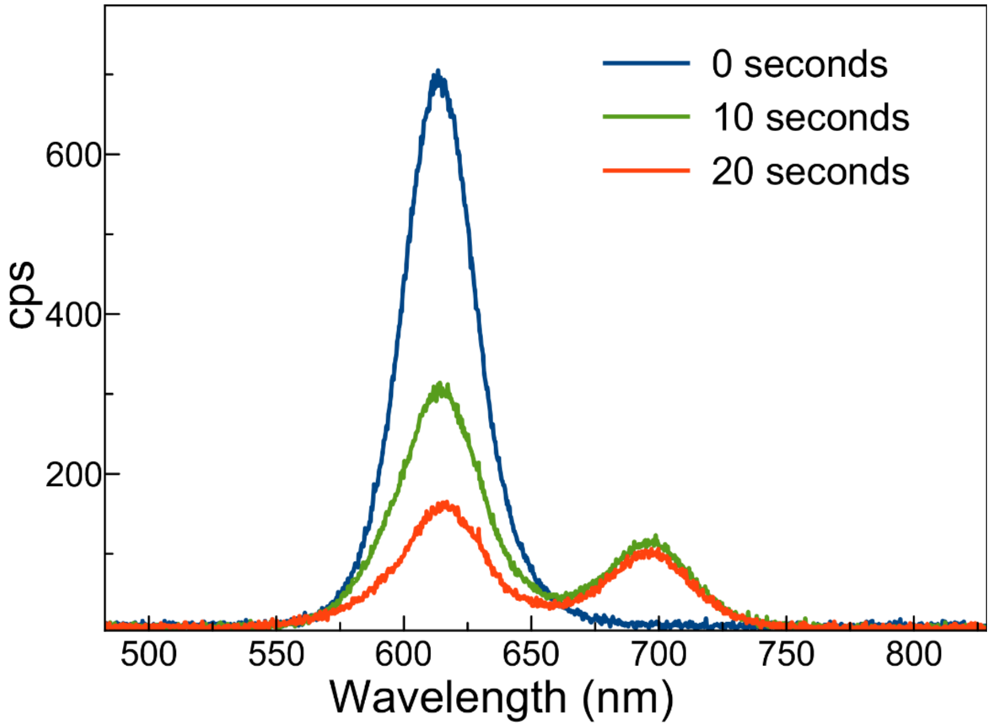

Supplementary Figure 8. Control experiment on a $CsPb(I_{0.5}Br_{0.5})_3$ nanocrystal-based film showing phase separation at I_{exc}=500 W cm^{-2}. Emission (cps, counts per second spectral changes of a $CsPb(I_{0.5}Br_{0.5})_3$ nanocrystal-based film at I_{exc}=500 W cm^{-2}.

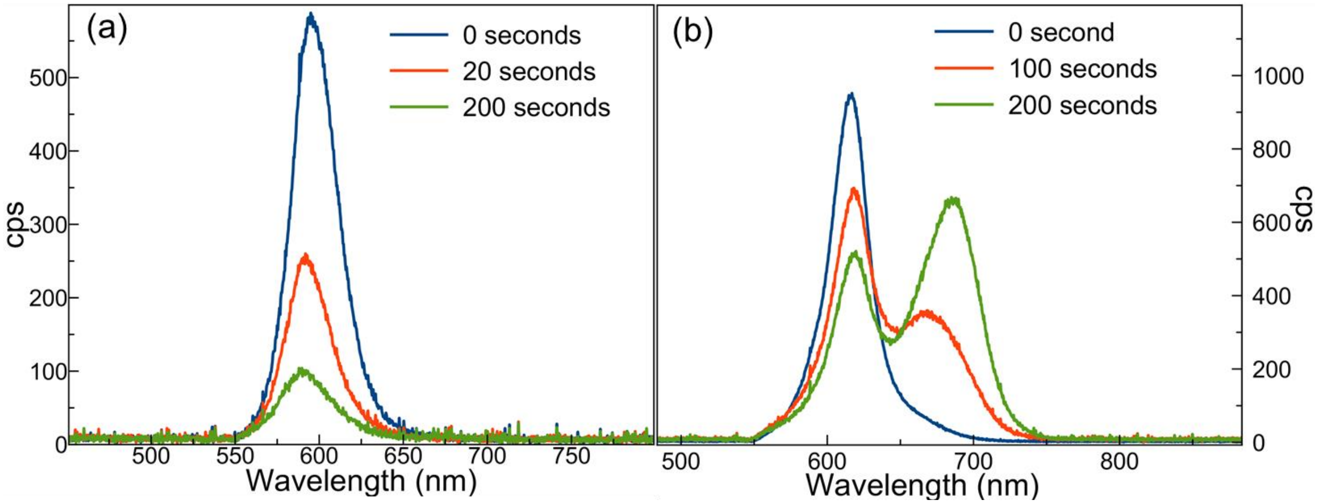

Supplementary Figure 9. Control experiment on a sintered $CsPb(I_{0.5}Br_{0.5})_3$ nanocrystal film. Emission (cps, counts per second) spectral changes observed in a thermally-treated $CsPb(I_{0.5}Br_{0.5})_3$ nanocrystal-based film at (a) I_{exc}=2.5 W cm^{-2} and (b) I_{exc}=55 W cm^{-2}.

	%I	%Br
Trial 1	54.3	45.7
Trial 2	55.8	44.2
Trial3	54.5	45.5
Average	54.9	45.1
I:Br	1.65	1.35

Supplementary Table 1: Energy Dispersive X-Ray Spectroscopy of mixed halide nanocrystals.

Supplementary Note 1. Microscopic phase separation model.

Preliminaries

The total fraction of mixed phase (ϕ_{mix}), iodide-rich (ϕ_{I}) and bromide-rich (ϕ_{Br}) domains in the thin film is given by

$$1 = \phi_{\mathrm{mix}} + \phi_{\mathrm{I}} + \phi_{\mathrm{Br}}.$$

Since $\phi_{\mathrm{I}} = \phi_{\mathrm{Br}}$ (halide segregation simultaneously produces iodide- and bromide-rich domains) one equivalently has

$$1 = \phi_{\mathrm{mix}} + 2\phi_{\mathrm{I}} = \phi_{\mathrm{mix}} + \phi_{\mathrm{ps}}$$

where ϕ_{ps} is fraction of phase-separated domains ($\phi_{\mathrm{Br}} + \phi_{\mathrm{I}}$). Solving for ϕ_{mix} then gives

$$\phi_{\mathrm{mix}} = 1 - \phi_{\mathrm{ps}} \ . \tag{1}$$

Relevant rate processes

The following are relevant kinetic rate processes involving the hybrid perovskite during photoexcitation.

Process 1. Carrier generation

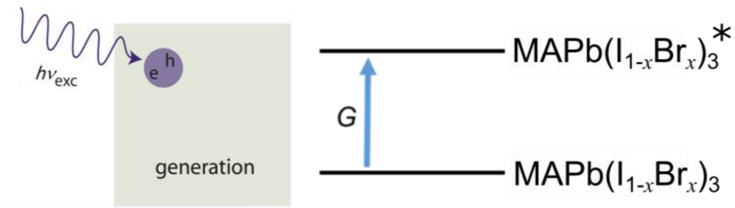

Supplementary Figure 10. Charge generation in $MAPb(I_{1-x}Br_x)_3$ (MA=$CH_3NH_3^+$).

The excitation rate (G) of the hybrid perovskite with incident light is given by

$$G = \frac{I_{\mathrm{exc}}\alpha}{h\nu_{\mathrm{exc}}} \tag{2}$$

where I_{exc} is the excitation intensity (W cm^{-2}), α is the hybrid perovskite absorption coefficient (cm^{-1}), and $h\nu_{\mathrm{exc}}$ is the energy of the incident photon (J photon^{-1}). The corresponding photogenerated carrier density is

$$\rho = G\tau \tag{3}$$

where τ is the carrier lifetime.

Process 2. Direct recombination yielding emission from the mixed halide perovskite

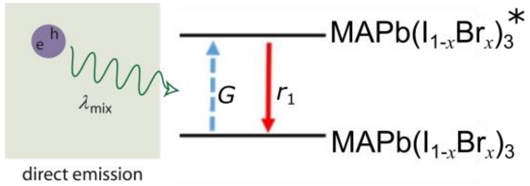

Supplementary Figure 11. Radiative recombination in $MAPb(I_{1-x}Br_x)_3$ (MA=$CH_3NH_3^+$).

The associated emission rate from the mixed halide perovskite is expressed as

$$r_1 = QY\phi_{\mathrm{mix}}G = QY\left(1 - \phi_{\mathrm{ps}}\right)G \tag{4}$$

where QY is the perovskite's external quantum yield, which for simplicity is assumed to be a constant over all excitation intensities.

Process 3. Phase segregation following excitation

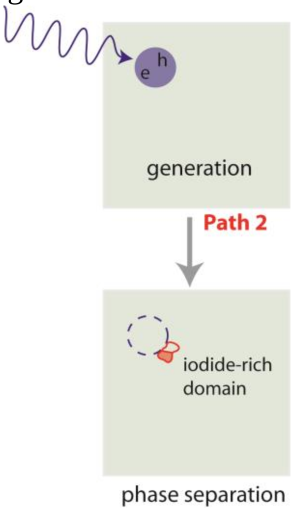

Supplementary Figure 12. Light-induced formation of iodide-rich domains.

We take the phase separation rate to be first order for a given excitation intensity G

$$r_2 = k_2 \phi_{\text{mix}} G = k_2 (1 - \phi_{\text{ps}}) G. \quad (5)$$

In the equation, k_2 is a unitless probability associated with inducing phase segregation upon excitation. We treat the I_{exc}-dependence of k_2 below.

Process 4. Phase segregation recovery

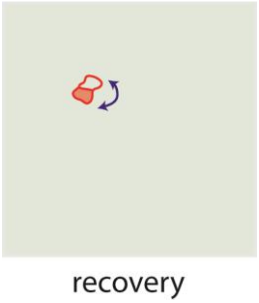

Supplementary Figure 13. Phase segregation recovery.

We treat the phase segregation recovery as first order in domain concentration:

$$r_3 = k_3 \phi_{\text{ps}} n \quad (6)$$

where n is the average molar volume of the domains.

Phase segregated fraction, ϕ_{ps}

Processes 3 and 4 contribute to changes in the phase segregated fraction, ϕ_{ps}. Consequently, the total rate of change of ϕ_{ps} can be expressed as

$$n\frac{d\phi_{\mathrm{ps}}}{dt} = r_2 - r_3.$$

Using Supplementary Equations 5 and 6 for r_2 and r_3 we therefore have

$$\frac{d\phi_{\mathrm{ps}}}{dt} = k_2(1-\phi_{\mathrm{ps}})\frac{G}{n} - k_3\phi_{\mathrm{ps}} = c_1 - c_2\phi_{\mathrm{ps}}$$

where $c_1 = k_2\frac{G}{n}$ and $c_2 = c_1 + k_3$. The solution to this differential equation with the initial condition $\phi_{\mathrm{ps}}(0) = 0$ is

$$\phi_{\mathrm{ps}} = \frac{c_1}{c_1+c_2}(1-e^{-c_2 t}). \quad (7)$$

In the limit that photo-induced phase separation occurs on a significantly faster timescale than thermal recovery

$$\boxed{\phi_{\mathrm{I}} = \frac{1}{2}\phi_{\mathrm{ps}} \approx \frac{1}{2}(1-e^{-c_2 t}).} \quad (8)$$

This is Equation 2 of the main text when $c_1 \gg k_3$.

Connection to the emission rate

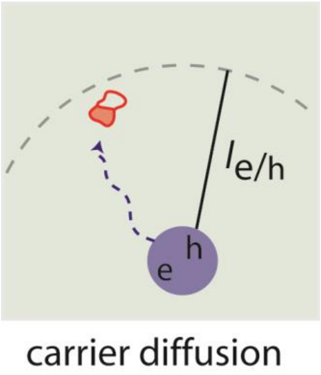

Supplementary Figure 14. Carrier diffusion to iodide-rich domains.

The rate of appearance of the iodide-rich phase (Process 3) is much slower than the rate at which the iodide-rich photoluminescence (I_{iodide}) changes. Thus, we posit that the largest contribution to I_{iodide} comes from the migration of carriers generated in the parent mixed halide phase to nearby

iodide-rich regions, resulting in subsequent radiative recombination. Note that our DFT calculations below suggest hole diffusion as the dominant process taking place due to the existence of favorable valence band offsets between $MAPb(I_{1-x}Br_x)_3$ and iodide-rich regions, but the exact nature of the carriers is immaterial to the basic arguments.

The underlying assumption is that the presence of an iodide-rich region within the geometric volume defined by $l_{\mathrm{e/h}}$ means that charge migration dominates direct photogenerated carrier recombination (Process 2). Plausibility for this can be seen by comparing the time for charge diffusion τ_{d} to the carrier lifetime (τ) as determined from experimental emission decay measurements. Namely, using the Einstein relation ($\tau_{\mathrm{d}} = \frac{{l_{\mathrm{e/h}}}^2}{D}$) with $l_{\mathrm{e/h}}$=100 nm and a diffusion coefficient of D=0.08 cm^2 s^{-1} [1] we obtain τ_{d}=1.25 ns which is significantly shorter than τ which is on the order of 62 ns.[1]

Consequently, I_{iodide} is proportional to the Poisson distributed probability (P) of carriers encountering at least one iodide domain upon diffusion. For analytical tractability, this probability can be expressed in terms of the probability of encountering no iodide-rich domain (P_{none}) and is

$$P = 1 - P_{\mathrm{none}} \tag{9}$$

where $P_{\mathrm{none}}=e^{-n_{\mathrm{I}}}$ with n_{I} the average number of iodide domains within the diffusion volume (V_{D}). We therefore obtain the following expression for $I_{\mathrm{iodide}} \propto P$

$$I_{\mathrm{iodide}} = I_{\mathrm{sat}}(1 - e^{-n_{\mathrm{I}}}) \tag{10}$$

with I_{sat} an empirical saturation photoluminescence. In the equation, n_{I} can be expressed in terms of V_{D}, the average volume of iodide-rich domains ($\bar{V}$), and ϕ_{I} such that

$$\frac{I_{\mathrm{iodide}}}{I_{\mathrm{sat}}} = 1 - e^{\frac{V_{\mathrm{D}}}{\bar{V}}\phi_{\mathrm{I}}}. \tag{11}$$

Using the linear approximation of Supplementary Equation 8 then gives

$$\phi_{\mathrm{I}} \approx \frac{1}{2} k_2 \left(\frac{G}{n}\right) t \tag{12}$$

from where we obtain the following expression for $I_{\mathrm{iodine}}/I_{\mathrm{sat}}$

$$\frac{I_{\mathrm{iodine}}}{I_{\mathrm{sat}}} \approx \left[1 - \exp\left(-\frac{V_{\mathrm{D}}}{\bar{V}} \frac{k_2}{2} \frac{G}{n} t\right)\right]. \tag{13}$$

Finally, defining an effective (experimental) forward rate constant as

$$k_{\mathrm{forward,em}} = \frac{V_{\mathrm{D}}}{2\bar{V}} \frac{G}{n} k_2$$

yields

$$\boxed{\frac{I_{\text{iodide}}}{I_{\text{sat}}} \approx 1 - \exp(-k_{\text{forward,em}}t).} \quad (14)$$

which is Equation 3 of the main text.

Supplementary Note 2. DFT calculations, band alignment and estimate of $n_{\max}$

We use DFT to estimate the ground state formation energy of $MAPb(I_{1-x}Br_x)_3$, $MAPbBr_3$, and $MAPbI_3$. The resulting ground state formation difference per formula unit is defined as

$$\Delta E_{\text{GS}}(x) = E_x - xE_{\text{Br}} - (1-x)E_{\text{I}},$$

where E_x, E_{Br} and E_{I} are the GGA energies per formula units of $MAPb(I_{1-x}Br_x)_3$, $MAPbBr_3$, and $MAPbI_3$, respectively. We use the experimentally observed cubic structure for $MAPbBr_3$ (symmetry $Pm\overline{3}m$ [2]) with a GGA-computed lattice constant of 6.14 Å. We compute the energies of $MAPbI_3$ and the mixed halides starting from the orthorhombic structure (symmetry Pnma (Z=4) [3,4]). The GGA-computed lattice constants of the iodide orthorhombic phase are 9.25 × 12.89 × 8.62 Å. We choose orthorhombic over tetragonal across the compositional domain for computational convenience, because it avoids orientational ambiguity for MA cations in the tetragonal structure.

To model the mixed halide, we start from four formula unit cells of iodide, containing 12 iodide ions, generate mixed configurations by replacing iodide with bromide, and relax the orthorhombic cell parameters and fractional coordinates. We consider six compositions ranging from x = 1/12 to ½ in 1/12 steps. The configurations are chosen arbitrarily to maximize the separation between the bromide ions and to probe the first-nearest-neighbor interactions that would be expected to dominate any ordering tendencies. A representative structure at x = 0.5 is shown in Supplementary Figure 4. CONTCARs of all relaxed structures are included as separate files.

Computed ΔE_{GS} values per two formula units, or FU, are shown in Supplementary Figure 5a as solid blue circles. Solid lines are included as a guide to the eye. From the plot it is evident that $MAPb(I_{1-x}Br_x)_3$ formation energies are small and positive up to $x \approx 1/3$, above which formation energies become negative. These results can be compared with the GGA results of Brivio *et al.* [5], who employed a more restrictive tetragonal representation of mixed halide structures but considered a greater variety of anion configurations at a given composition. Brivio *et al.* [5] found formation energies to be similarly positive and of order 0.01 eV formula unit^{-1} to even higher compositions.

Both the results reported here and those of Brivio *et al.*[5] are based on modest-sized computational cells that imply some level of ordering and do not consider the consequences of MA dynamics. Consequently, we cannot rule out the existence of much lower energy structures when these assumptions are relaxed. However, there is no evidence for this possibility either in the DFT calculations or experimental observations. Thus, all available DFT data are consistent with ground state ΔE_{GS} formation energies that approach zero in the iodide-rich region (i.e. x less than 0.5).

Next, to account for entropic effects on free energies of formation, we combine $\Delta E_{\mathrm{GS}}(x)$ with the ideal entropy of mixing on the anion sub-lattice. Resulting free energies of formation per formula unit ($\Delta F_{\mathrm{GS}}(x)$) are then

$$\Delta F_{\mathrm{GS}}(x) = \Delta E_{\mathrm{GS}}(x) - 3k_{\mathrm{B}}T\,[x \ln x + (1-x)\ln(1-x)]. \quad (15)$$

Supplementary Figure 5a shows this formation free energy evaluated at 300 K as closed green squares connected with a line to guide the eye. $\Delta F_{\mathrm{GS}}(x)$ is negative over the entire composition range of interest, consistent with an entropy-driven preference for mixing on the halide sublattice at relevant temperatures. Brivio *et al.* [5] evaluated mixed state free energies using a somewhat more sophisticated quasi-chemical approximation to arrive at similar conclusions of an entropy-driven preference for mixing.

$MAPb(I_{1-x}Br_x)_3$ and $MAPbI_3$ relative band alignment. Relaxed structures extracted from the above DFT calculations enable computational estimates of $MAPb(I_{1-x}Br_x)_3$, $MAPbI_3$, and $MAPbBr_3$ valence band energies. Informing theory are experimental ultraviolet photoelectron spectroscopy (UPS) measurements that indicate that the conduction band edges of $MAPbI_3$ and its mixed halide analogues are dominated by Pb 6p states and are approximately isoenergetic. In contrast, valence band states are dominated by contributions from halide anion states and hence are more sensitive to composition.[6,7] Supplementary Figure 5c shows resulting DFT-computed valence band energies along with associated conduction band energies.

The results show that $MAPbBr_3$ band offsets are unfavorable for either electron or hole localization within bromide-rich regions of hybrid perovskite films. We consequently focus on band offsets present at the $MAPb(I_{1-x}Br_x)_3$ and $MAPbI_3$ interface. We construct an orthorhombic supercell doubled in the long direction, consisting of two layers of mixed perovskite below two layers of $MAPbI_3$. Lateral lattice constants of the supercell match those of $MAPbI_3$. The mixed domain is constructed by replacing every other iodide ion with bromide. After relaxing atomic positions, the layer-by-layer projected density of states is computed and is plotted in Supplementary Figure 5c. In agreement with experimental inferences, we find that $MAPb(I_{1-x}Br_x)_3$ and $MAPbI_3$ conduction band edges are essentially isoenergetic and that the valence band maximum of $MAPbI_3$ is 0.2 eV above that of $MAPb(I_{0.5}Br_{0.5})_3$ (Supplementary Figure 5c). The GGA-computed band gap is also consistent with experiment, likely due to cancellation of relativistic and many-body effects.[8]

Because the conduction bands of $MAPb(I_{1-x}Br_x)_3$ and $MAPbI_3$ are effectively isoenergetic, their valence band offsets across the compositional range of interest can be captured using known experimental band gap differences. The band gap is a function of the composition *x*, increasing from 1.57 eV in the iodide form to 2.29 eV in the bromide form.[9] Noh *et al.*[9] have fit the experimentally-observed band gap (E_{g}) difference (ΔE_{g}) between $MAPb(I_{1-x}Br_x)_3$ (E_x^{g}) and $MAPbI_3$ ($E_{\mathrm{I}}^{\mathrm{g}}$) phase as a function of composition according to:

$$\Delta E_{\mathrm{g}}(x) = E_x^{\mathrm{g}} - E_{\mathrm{I}}^{\mathrm{g}} = 0.39x + 0.33x^2 \mathrm{eV}.$$

ΔE_{g} increases monotonically with *x* and reaches 0.28 eV at $x = 0.5$, for instance. This monotonic trend is consistent with the preferential localization of photogenerated holes in iodide-rich regions of hybrid perovskite film upon photoexcitation.[10]

Optical excitation

Next, we consider how this band gap difference alters the thermodynamic preference for phase separation when a $MAPb(I_{1-x}Br_x)_3$ sample is under illumination. We estimate this effect by evaluating the free energy of complete phase separation in the presence of a photo-generated hole:

$$n\mathrm{MAPb}(\mathrm{Br}_x\mathrm{I}_{1-x})_3^* \rightarrow nx\mathrm{MAPbBr}_3 + n(1-x)\mathrm{MAPbI}_3^*.$$

Here n represents some number of formula units. We write the free energy of this process as

$$\Delta F^*(x,n) = n(F^*_{\mathrm{mix}}(x) - x\,F_{\mathrm{Br}} - F^*_{\mathrm{I}}) = n\Delta F_{\mathrm{GS}}(x) + \Delta E_{\mathrm{g}}(x) \tag{16}$$

where F^*_{mix}, F_{Br}, and F^*_{I} are free energies of photoexcited $MAPb(I_{1-x}Br_x)_3$, $MAPbBr_3$, and $MAPbI_3$. This is Equation 1 of the main text. Supplementary Figure 5a shows the resulting ΔF^* computed for n = 2 formula units at 300 K. The excitation energy difference is greater than the mixing free energy and is substantially greater than the DFT-computed mixing energy. Thus, optical excitation carries sufficient energy to drive multiple formula units of the mixed phase into bromide- and iodide-rich phases, supporting the kinetic model.

Supplementary Equation 16 is written for separation into pure domains. More precisely, the separated domain compositions will be a compromise between how the free energies and the band gap vary with composition, how these are influenced by interfacial energies, and will even be influenced by the dynamics of anion mobility. Taking decomposition into pure phases yields the maximum ΔF_{GS} and thus the free energy cost of phase separation. Separation into iodide- and bromide-enriched phases will yield correspondingly smaller ΔF_{GS} values. Supplementary Figure 5a shows ΔF^* to be near zero in the domain x less than 0.1 with a predominantly iodide-rich phase composition of $MAPb(I_{0.9}Br_{0.1})_3$. The estimate is consistent with the results of McGehee *et al.* [11] who have previously suggested that iodide-rich phases following prolonged photoexcitation adopt a composition with x approximately 0.2 [i.e. $MAPb(I_{0.8}Br_{0.2})_3$].

Finally, we can estimate the initial domain size of phase-separated regions. The free energy cost of phase separating is balanced by the energy advantage of localizing a charge carrier in an iodide-rich region when Supplementary Equation 16 equals zero. Solving for n yields

$$n_{\mathrm{max}}(x) = -\frac{\Delta E_{\mathrm{g}}(x)}{\Delta F_{\mathrm{GS}}(x)} \tag{17}$$

which is Equation 2 of the main text. Supplementary Figure 5b shows n_{max} obtained at three different temperatures with predicted values on the order of several formula units. Note, however, that because the ideal mixing free energy contribution varies most rapidly near the composition limits while the band gap difference does not, the denominator of Supplementary Equation 17 will be smaller and n_{max} larger if the system separates into less than 100% bromide and iodide domains (i.e. iodide- and bromide-enriched domains). This result and the ΔF^* estimates shown previously in Supplementary Figure 5a thus all point to the important role played by band gap differences, between the parent mixed halide and iodide-enriched domains, in driving phase segregation under optical illumination.

Supplementary Note 3. Intensity dependence of the phase segregation rate constant

The non-linear I_{exc}-dependence of $k_{\mathrm{forward,em}}$ (Figure 1d, main text) indicates that it is a non-linear function of photogenerated carrier density (ρ). This observed dependence is readily

rationalized using a simple model where the probability of creating a phase-separated domain within the geometric volume defined by the carrier diffusion length ($l_{e/h}$) is the same irrespective of the number of carriers in that volume. To this end, we assume that $k_{forward,em}$ is proportional to the Poisson-distributed probability that at least one carrier exists within V_D. For analytical tractability, this probability is expressed in terms of the probability for having no carrier within V_D (P_{none}) so that

$$k_{\text{forward,em}} \propto 1 - P_{\text{none}}. \tag{18}$$

Next, since the average number of carriers within V_D is $V_D\rho$ we express P_{none} as $P_{\text{none}} = e^{-V_D\rho}$ so that

$$\boxed{k_{\text{forward,em}} = k_{\text{sat}}(1 - e^{-V_D\rho}) = k_{\text{sat}}\left(1 - e^{-\beta I_{\text{exc}}}\right).} \tag{19}$$

where k_{sat} is an empirical saturation rate constant at large excitation intensities and β is an empirical fitting parameter. This is Equation 4 of the main text.

Note that Supplementary Equation 19 can be used as a check for the model's self-consistency. Namely, if Supplementary Equation 18 is fit to the experimental data in Figure 1d of the main text, we obtain $k_{\text{sat}} = 0.7\ \text{s}^{-1}$ and $\beta = 0.29$ cm^2 mW^{-1}. From $\beta = \frac{V_D\rho}{I_{\text{exc}}} = \frac{V_D\alpha\tau}{h\upsilon_{\text{exc}}}$ with $\rho = G\tau$ (Supplementary Equation 3), $h\nu_{exc}$ = 4.8 eV photon^{-1} and the following order of magnitude values: τ=10 ns and $\alpha = 10^5$ cm^{-1}, we find an associated diffusion length of $l = 320$ nm, which agrees well with known literature values of $l_{e/h}$~100 nm.[12]

At sufficiently low I_{exc}-values, the phase segregation recovery rate exceeds phase separation. Consequently, Supplementary Equation 19 allows us to estimate an excitation intensity threshold (I_{th}) below which phase segregation is unfavored. We assume this to occur when $k_{forward,em}$ is an order of magnitude smaller than $k_{reverse,em}$. Using $k_{reverse,em}$=6.8×10^{-3} s^{-1}, $k_{\text{sat}} = 0.7$ s^{-1} and $\beta = 0.29$ cm^2 mW^{-1}, we estimate an intensity threshold of I_{th} ~ 20 μW cm^{-2}, which agrees well with the experimentally-determined threshold value of I_{th} approximatively 40 μW cm^{-2}.

Supplementary Note 4. Synthesis of $CsPb(I_{1-x}Br_x)_3$ perovskite nanocrystals and thin films.

Mixed halide perovskites were synthesized using a previously reported procedure[13] with slight modifications. Cs-oleate was synthesized by combining Cs_2CO_3 (0.814g, Sigma-Aldrich 99.9%), 2.5 mL of oleic acid (Sigma-Aldrich, 90%), and 40 mL of octadecene (Sigma-Aldrich, 90%) in a 100 mL 3-neck flask. The reaction mixture was then degassed at 120 °C for 1 hr. Following degassing, the vessel was backfilled with N_2 and was heated to 150 °C. The resulting solution was used without any further purification.

Mixed halide perovskite nanocrystals were synthesized by adding the desired molar ratio of $PbBr_2$ (Sigma-Aldrich, 99.999%) to PbI_2 (Sigma-Aldrich, 99.999%) to 5 mL of octadecene in a 10 mL 3-neck flask. The mixture was degassed under vacuum for 1 hr at 120 °C and was then backfilled with N_2. Then, 1 mL of oleylamine (Acros, 80-90%) and 1 mL of oleic acid were injected into the mixture. After the lead salts had dissolved completely, the reaction mixture was

heated to 170 °C whereupon 1 mL of the Cs-Oleate precursor was injected. Note that the Cs-oleate precursor falls out of solution at room temperature. Consequently, the injection mixture was heated to 110 °C prior to introduction into the reaction vessel. The reaction was immediately quenched using an ice bath whereupon nanocrystals were isolated by centrifugation at 7000 rpm for 10 minutes. The supernatant was discarded and the resulting pellet was rinsed with a 1:5 mixture of acetone to toluene. The product was then dispersed in hexanes for further characterization.

$CsPb(I_{1-x}Br_x)_3$ perovskite thin films were synthesized using a previously reported procedure.[14] All manipulations were conducted in a nitrogen atmosphere glovebox. Equal molar amounts of CsBr (Sigma-Aldrich, 99.999%), CsI (Sigma-Aldrich, 99.999%), $PbBr_2$ and PbI_2 were dissolved in dry dimethyl sulfoxide (DMSO) in order to obtain a 1M $CsPbI_{1.5}Br_{1.5}$ perovskite thin film precursor solution. The precursor solution was then spin coated onto cleaned cover glass (Vetrini Coprioggetto) at 6000 rpm. The films were subsequently annealed at 60 °C for 2 minutes. In the thermal annealing control experiment, nanocrystal-based films were subjected to an additional annealing step at 170 °C for 5 minutes.

Supplementary references

[1] Guo, Z., Manser, J.S., Wan, P., Kamat, P.V. & Huang, L. Spatial and temporal imaging of long-range charge transport in perovskite thin films by ultrafast microscopy. *Nat. Commun.* **6**, 1–7 (2015).

[2] Poglitsch, A. & Weber, D. Dynamic disorder in methylammoniumtrihalogenoplumbates (II) observed by millimeter-wave spectroscopy. *J. Chem. Phys.* **87,** 6373 (1987).

[3] Ong, K. P., Goh, T. W., Xu, Q. & Huan, A. Mechanical origin of the structural phase transition in methylammonium lead iodide $CH_3NH_3PbI_3$. *J. Phys. Chem. Lett.* **6,** 681–685 (2015).

[4] Ong, K. P., Goh, T. W., Xu, Q. & Huan, A. Structural Evolution in Methylammonium Lead Iodide $CH_3NH_3PbI_3$. *J. Phys. Chem. A* **119,** 11033–11038 (2015).

[5] Brivio, F., Caetano, C. & Walsh, A. Thermodynamic origin of photoinstability in the $CH_3NH_3Pb(I_{1-x}Br_x)_3$ hybrid halide perovskite alloy. *J. Phys. Chem. Lett.* **7,** 1083–1087 (2016).

[6] Butler, K. T., Frost, J. M. & Walsh, A. Band alignment of the hybrid halide perovskites $CH_3NH_3PbCl_3$, $CH_3NH_3PbBr_3$ and $CH_3NH_3PbI_3$. *Mater. Horizons* **2,** Advance (2015).

[7] Bretschneider, S. A., Weickert, J., Dorman, J. A. & Schmidt-Mende, L. Research update: Physical and electrical characteristics of lead halide perovskites for solar cell applications. *APL Mater*. **2**, 040701 (2014).

[8] Umari, P., Mosconi, E. & De Angelis, F. Relativistic GW calculations on $CH_3NH_3PbI_3$ and $CH_3NH_3SnI_3$ perovskites for solar cell applications. Sci. Rep. 4, 4467 (2014).

[9] Noh, J. H., Im, S. H., Heo, J. H., Mandal, T. N. & Seok, S. Il. Chemical management for colorful, efficient, and stable inorganic-organic hybrid nanostructured solar cells. *Nano Lett.* **13,** 1764–1769 (2013).

[10] Yoon, S. J. *et al.* Tracking iodide and bromide ion segregation in mixed halide lead perovskite during photoirradiation. *ACS Energy Lett.* **1,** 290-296 (2016).

[11] Hoke, E. T. *et al.* Reversible photo-induced trap formation in mixed-halide hybrid perovskites for photovoltaics. *Chem. Sci.* **6,** 613–617 (2015).

[12] Sum, T. C. & Mathews, N. Advancements in perovskite solar cells: Photophysics behind the photovoltaics. *Energy Environ. Sci.* 7, 2518–2534 (2014).
[13] Protesescu, L. *et al.* Nanocrystals of cesium lead halide perovskites ($CsPbX_3$, X = Cl, Br, and I): novel optoelectronic materials showing bright emission with wide color gamut. *Nano Lett.* **15,** 3692–3696 (2015).
[14] Beal, R. E. *et al.* Cesium lead halide perovskites with improved stability for tandem solar cells. *J. Phys. Chem. Lett.* **7,** 746–751 (2016).
